# A comprehensive review on global contributions and recognition of pharmacy professionals amidst COVID-19 pandemic: moving from present to future

**DOI:** 10.1186/s43094-021-00273-9

**Published:** 2021-06-11

**Authors:** Saad Ahmed Sami, Kay Kay Shain Marma, Agnila Chakraborty, Tandra Singha, Ahmed Rakib, Md. Giash Uddin, Mohammed Kamrul Hossain, S. M. Naim Uddin

**Affiliations:** Department of Pharmacy, University of Chittagong, Chittagong, 4331 Bangladesh

**Keywords:** COVID-19, Pharmacist, Community, Hospital, Clinical, Industrial, Collaboration, Recognition

## Abstract

**Background:**

COVID-19, a respiratory tract infection caused by SARS-CoV-2, is a burning question worldwide as it gives rise to a pandemic situation. No specific medications are still recommended for COVID-19; however, healthcare support is crucial for ameliorating the disease condition. Pharmacists are the frontline fighters who are responsible for providing healthcare support to the COVID-19 infected patients around the world. This review endeavored to briefly rationalize the contributions of several pharmacy professionals in diverse fields along with their collaborative efforts and dedication regarding their limitations during the COVID-19 situation and view the prospects of pharmaceutical care services in the post-pandemic period.

**Main body of the abstract:**

Online databases were utilized to search for scholarly articles and organizational websites, to sum up the information about the contemporary and expanded role of pharmacists. Key articles were retrieved from Google Scholar, PubMed, and Science Direct databases using terms: “COVID-19,” “novel coronavirus,” “community,” “industrial,” “hospital,” “clinical,” “recognition,” “obstacles,” “collaboration,” “SARS-CoV-2,” “healthcare,” and “outbreak” in combination with “pharmacist.” The articles were included from the inception of the pandemic to January 25, 2021. The current review found pharmacist’s global contributions and involvements with other professionals to provide healthcare services amidst COVID-19. This included testing of suspects, providing medical information, psycho-social support, debunking myths, mitigating drug shortage events, telemedicine, e-prescription, infection control, and controlling the drug supply chain. In many countries, pharmacists’ activities were much appreciated but in some countries, they were not properly acknowledged for their contributions amidst COVID-19 outbreak. They played additional roles such as participating in the antimicrobial stewardship team, improving value-added services, conducting clinical data analysis to suppress the outspread of the SARS-CoV-2.

**Short conclusion:**

During the COVID-19 pandemic while the whole world is fighting against an invisible virus, the pharmacists are the earnest hero to serve their responsibilities along with additional activities. They need to be prepared and collaborate with other healthcare professionals further to meet the challenges of post-pandemic circumstances.

## Background

The world has witnessed unprecedented outbreaks of many life-threatening viral diseases caused by an array of pathogenic organisms in the last few decades including Ebola, SARS (severe acute respiratory syndrome), MERS (Middle East respiratory syndrome), Chikungunya, and Influenza. In late December of 2019, a cluster of febrile respiratory cases greatly resembling viral pneumonia with unknown etiology was reported by the Chinese authorities. The metagenomic RNA sequencing of the pathogenic agent and the bronchoalveolar lavage fluid samples isolated from the patients identified a novel strain of human beta-coronavirus designated as severe acute respiratory syndrome coronavirus 2 (SARS-CoV-2). The clinical condition caused by this causative agent is referred to as COVID-19 [1]. The spreading of the novel coronavirus (COVID-19) has been exponential that was not globally anticipated earlier. The devastating impact of this fatal outbreak has surpassed several viral infections we previously encountered and has become the center of global attention. Originating from Wuhan, one of the populous Chinese City of Hubei province in late December of 2019, this acute respiratory infection has deployed a significant toll on people across the globe in a short period and compelled the World Health Organization (WHO) to declare this outbreak as a pandemic on March 11, 2020 [2]. As of February 21, 2021, more than 110 million infected cases with over 2.4 million deaths have been reported worldwide [3].

COVID-19 is the third coronavirus outbreak of the twenty-first century and zoonotic origins like its predecessors (SARS, MERS). This virus can be transmitted via direct close contact and fomite transmission. Although symptoms generally appear but can also be asymptomatic that makes it even more dangerous. The symptoms of this viral infection may range from mild or moderate fever to severe pneumonia. The most common symptoms include combinations of fever, dry cough, loss of smell or taste, sore throat, and shortness of breath. Immunocompromised old-age patients with multi-organ failure and comorbidities are more susceptible and experienced the worse outcomes [4]. This viral infection has upended the global healthcare system by putting pressure on healthcare professionals and increasingly straining the supply of qualified healthcare providers. As COVID-19 is a contagious infection, there is a significant risk for healthcare professionals to be infected by this viral disease [5]. Watson and his team conducted a Delphi study that corroborated the competent role of pharmacy professionals in disaster management. Pharmacists can carry out their responsibility through direct or indirect patient contact and play a significant role in disease containment and management [6]. It has been well recognized that the commendable efforts and extraordinary performances were shown by the pharmacists in previous health and disaster crisis, for example, Zika, Ebola, H1N1 influenza, measles attack, anthrax crisis, and opioid crisis have made them an integral part of the healthcare team [7, 8]. A USA-based study reported that, more than 85% of public health programs involve pharmacists in the vaccine distribution plans during pandemic situation [9]. During the 2009 H1N1 influenza pandemic, it was considered a convenient place for many patients to receive vaccinations [10]. In the event of any global epidemic, 93 percent of pharmacists at that time were able to maintain their services [11]. In Australia, within just 2 years, pharmacists have administered more than 35,000 influenza vaccines and Canadian pharmacists provided over 765,000 vaccinations in their pharmacy premises [12]. Pharmacies have become the most commonly used non-medical setting for flu vaccination in adults due to convenience and lower cost [13].

Though pharmacists contributed to previous pandemics but this time it is more difficult than before as the novel coronavirus is intractable, no vaccine or therapeutic guideline found and it suddenly manifested even without any precautions taken earlier on. Amid the public health crisis of current magnitude, pharmacists are adopting innovative strategies and working at the forefront alongside physicians, nurses, paramedics, and non-technical staffs to combat the chaos attributed to the COVID-19 infection [14]. Being part of an interdisciplinary team, pharmacists play a vital role in helping patients and doctors interact better [15]. They remain the most reliable, accessible, and convenient professional for patients to provide medication service [16]. It has been over a year, pharmacists across the globe are carrying out their responsibilities for the betterment of the current situation. Pharmacists can play a potential role in this crisis time along with other healthcare workers.

**Fig. 1:**
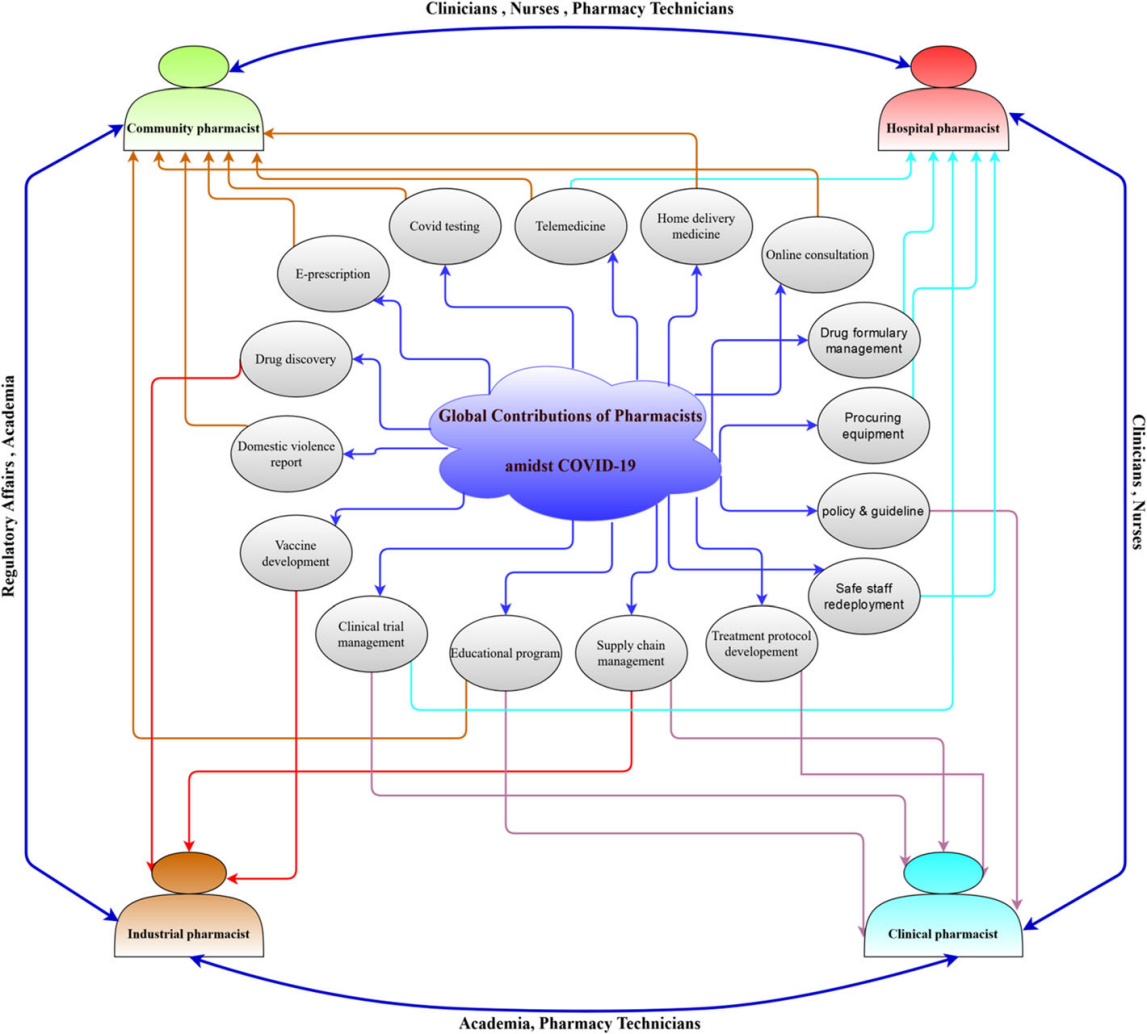
Overview of global contributions of pharmacists amidst COVID-19

In our current study, a literature search was performed to summarize the knowledge on the contribution of pharmacy professionals during the COVID-19 pandemic. The relevant articles were retrieved mainly from electronic databases including Google Scholar (https://scholar.google.com/), PubMed (https://pubmed.ncbi.nlm.nih.gov/), and Science Direct (https://www.sciencedirect.com/). We conducted our search using the following terms: “COVID-19,” “novel coronavirus,” “community,” “industrial,” “hospital,” “clinical,” “recognition,” “obstacles,” “collaboration,” “SARS-CoV-2,” “healthcare,” and “outbreak” in combination with “pharmacist.” The articles including implications of pharmaceutical care services during the COVID-19 period and relevant pre-pandemic data were also covered as much as possible. Original research articles, review articles, commentaries, editorials, letters to the editor, authentic guidelines on the management of novel coronavirus pandemic, and official web pages of different pharmaceutical organizations were included in this study from the inception of the pandemic to January 25, 2021. Poster papers, advertisements, thesis works, preprints, and published studies in non-English languages were excluded from this study. The available materials were assembled carefully to point out the contemporary and expanded role of pharmacists across the world during the COVID-19 outbreak. The purpose of this review is to describe the global contributions of pharmacy professionals in different settings, their collaborative efforts, worldwide recognitions, limitations during the COVID-19 situation, and prospects of the pharmaceutical care services in the post-pandemic period (Fig. 1).

## Contributions of community pharmacists in COVID-19

Community pharmacists are healthcare providers who play vital roles in responding to symptoms, supplying medicines, and providing health promotion in the communities where they serve. They are being recognized not only as health professionals but also educators and counselors of patients, mentors and researchers, creative business formulators and developers, managers and leaders, and stakeholders [17]. They are the most accessible healthcare provider and a primary focal point for consumers to receive reliable evidence-based information when there is no hospital available nearby [18]. Community pharmacists are making essential public health contributions in rural areas where patients have to face obstacles such as acute shortage of primary care providers without proper facilities, hazardous terrain, and lack of public transportation with extended time in traveling that force them to postpone the needed health services [13].

Pharmacy care services during COVID-19 were divided into two categories; one was prevention and control of the pandemic and the other was to provide pharmacy services to the patients. Community pharmacy management teams supported primary care services by providing an adequate supply of COVID-19 related medications and preventative products, following environment regulations, and providing sufficient staff training. Pharmacists have used various approaches to provide primary care services in drug dispensing, consulting and referrals, chronic disease management, safe use of infusions, patient education, home care guidance, and psychological support to promote the COVID-19 pandemic control and ensure safe medication use of community patients during the pandemic [19]. Community pharmacies were one of the few places which remained open 24 h daily amidst the heat of the total global lockdown to serve the populace [20]. During the current pandemic, it is recognized that community pharmacies will often be the first point of contact with the health system for individuals with COVID-19 related health concerns or who require reliable information and advice [14]. If an effective vaccine is developed to prevent COVID-19 infection, community pharmacists will be accessing healthcare providers to offer this immunization as evidenced by the growing number of patients receiving their influenza vaccine at local pharmacies. Pharmacists have been providing vaccinations services throughout the last few decades in the countries like the UK, New Zealand, Canada, and Portugal [21]. In the USA, community pharmacies are positioned as the second most utilized site for adult influenza vaccine administration after physician’s chambers [22]. Hoti et al. reported about 90% of community pharmacists having specific knowledge for the implementation of COVID-19-related preventative measures. This evidence can be backed up by Chin and his colleagues who described the active role of Canadian pharmacists in SARS outbreak [17].

Many countries are leveraging a range of community pharmacy services where pharmacists are playing a pivotal role in controlling community transmission by educating the public and clients about COVID-19 signs and symptoms, offering counseling on COVID-19 precautionary measures including practicing social distancing, maintaining strict hand hygiene, avoiding touching facial T-region (nose, eyes, mouth). They have taken the responsibility of keeping the record of contact-tracing history, disinfecting surfaces of pharmacy premises regularly, and also triaging at the community level for suspected COVID-19 cases [23, 24]. In many countries, community pharmacies have expanded service-hours amidst strict lockdown period intending to promote medication adherence, handle out-of-hours emergency supply requests, deterring misinformation, providing medical supply pharmaceutical products and devices (face masks, over-the-counter drugs, disinfectants), and thus reducing the burden of general practice, unnecessary hospital visits, and health expenditure [25–27]. Amariles et al. recently depicted the critical role of pharmacists in minimizing community transmission of the COVID-19 virus by ensuring appropriate detection, referral, and suspected case management [28].

The review of health services provided by the community pharmacists in different countries across continents amidst COVID-19 would make it more evident. In many developed countries (USA, Spain, France, Canada, Denmark, Italy, Netherlands, Scotland, and Germany), community pharmacists leveraged on information communication technology to minimize direct patient contact and deliver essential pharmaceutical care services [29]. Recently, a study in Macau has demonstrated the importance of community pharmacist’s participation in the COVID-19 management system, where they were given the charge of maintaining overall infection progress and government approach “The Guaranteed Mask Supply for Macao residents Scheme” [30]. In Saudi Arabia, pharmacists delivered medications by mail with continuous counseling to keep the patients safe at home [31]. In many European countries, community pharmacists took part in the domestic violence campaign where they had the authority to report complaints [32]. In African countries, the diagnostic testing for the COVID-19 infection is a massive challenge due to the fragile healthcare structure of the region [33]. But even in some of those countries, community pharmacists are executing their duties to the best of their abilities [20, 29, 34].

Community pharmacists have provided crucial services to tackle the COVID-19 health crisis in many countries across the continents which are described briefly in Table 1.

**Table 1:** Services provided by community pharmacists in tackling COVID-19 health crisis

Continents	Country	Community activities
North America	USA [32, 35, 36]	• COVID-19 testing sites in community pharmacies • Gave initial treatment to the patients with allergic symptoms, influenza and Group A Streptococcus. • Performed emergency refill and certain acts without the supervision of a physician, authorized to treat certain conditions, • Performed remote data entry and taking new orders • Mitigated the shortage events of life saving drugs
	Canada [36, 37]	• Daily screening of patients • Offered home delivery services to COVID-19 vulnerable patients • Extending prescriptions and prescribing certain medications alongside doctors
Europe	UK [17, 32]	• Carried out online consultation service for patients (Pharmadoctor eTool) • Provided psycho-social support and prescription-free supply of particular controlled medicine to the patients with home-delivery of medicines to the self- isolating patients • Ensured rapid COVID-19 testing service
	France [32, 38]	• Renewal of chronic treatment by community pharmacists • Rights to prepare hydroalcoholic gels in case of shortage • Continuous supply of essential medicine with psycho-social support • Reported the complaints of domestic violence
	Italy [32]	• Decree for pharmacists to give oxygen to the patients • Introduction of e-prescription and disinfectant products preparation • Continuous supply of essential medicine and home delivery service to vulnerable populations by Federfarma.
	Spain [29, 32, 39]	• Offered service to the victims of gender violence by requesting a “Mask 19” • Provided clinical screening and interventions • Tele-pharmacy services for chronic disease
	Netherlands [29, 32]	• Reported the complaints of domestic violence to the pharmacy using code word “Masker 19” • Tele-pharmacy services for chronic disease
Oceania	Australia [34, 36]	• Reviewed medication orders and verification • Acted as an “central information center” to provide COVID-19 related information • Extend the supply of medications for chronic diseases • Provided home delivery medication services
	New Zealand [36]	• Provided one-stop pharmacy service that included home-delivery service • Disseminated proper information on community transmission and hand-hygiene
Asia	China [25, 38]	• Maintained controlled environment in the community • Ensured regular medical supplies (masks, OTC drugs, disinfectants, thermometer, etc.) and home delivery services through technology • Patient counseling services and served as a custodian of patient safety • Identified probable infections and recorded patients demographic as well as personal information
	Jordan [40]	• Conducted seminars and preparing educational videos related to the COVID-19 infection • Preparation of alcohol-based disinfectants
	Taiwan [41]	• Proper system for surgical masks distribution and internet based system to check the availability • Provided dispensing services to minimize prescription seeking patients
	Macao [30]	• Maintained overall infection progress and government approach “The Guaranteed Mask Supply for Macao residents Scheme”
	Saudi Arabia [31]	• Ensured proper use of medicine to prevent misuse of drugs. • Reported to concerned authority about any suspected case of COVID-19 • Developed informational content such as banner, poster, leaflet to facilitate MOH guideline about COVID-19 among people • Provided home delivery of medicine, telemedicine service and counseling to home quarantined individuals
	India [42, 43]	• Collected person sample from door to door • Identified patients with symptoms and send them to quarantine • Provided medications and supplies to their communities
	Pakistan [29, 38]	• Delivered pharmaceutical care through tele-pharmacy to the patient with chronic disease
	Qatar [34]	• Reviewed medication orders and verification • Participated in COVID-19 screening and testing • Provided home delivery of medications
South America	Colombia [28]	• Detection and appropriate referral of possible COVID-19 cases • Provided patient education
	Brazil [44]	• Provided information on COVID-19 • Review of medication history, drug therapy and follow-up • Medication adherence guidance in acute and chronic disease
Africa	Nigeria [20, 34]	• Debunked myths of using chloroquine and other unapproved drugs for the cure of COVID-19 • Reviewed medication orders and verification
	Egypt [29]	• Delivered pharmaceutical care through tele-pharmacy to the patient with chronic disease
	South Africa [34]	• Extend the supply of medications for chronic diseases • Reviewed medication orders and verification
	Kenya [20]	• Advocated zero-tax implementation on drugs to ensure drug affordability

## Contributions of hospital pharmacists in COVID-19

The ongoing COVID-19 pandemic has increased the responsibility of hospital pharmacists as they had to take care of the COVID-19 patients in hospitals and control the pandemic emergency. Their role cannot be overlooked as they directly contributed to COVID-19 management protocols alongside ICU (intensive care unit) nurses, physicians, and respiratory therapists. Participating in the antimicrobial stewardship programs makes them directly involved in planning and responding to pathogen outbreaks. These responsibilities also help them in determining the safety and effectiveness of new antiviral drug treatment [45]. They supported pharmaceutical care services and participated in the COVID-19 medical collaborative team to facilitate pandemic control by developing strategies to tackle drug shortages, ensuring the safe transition of care via medication reconciliation, reviewing treatment charts, and giving emotional support to the comorbid patients. Besides, they are disseminating information on prevention, detection, treatment of managing COVID-19 infections, and offering continuous healthcare service to patients with chronic diseases in hospitals. They have also combined clinical guidance and clinical research to help physicians in formulating drug regimens [46–49]. In Middle East and South Asia, pharmacy workflow was redesigned in many hospitals. Outpatient pharmacies of some hospitals removed many chairs from the waiting room areas to promote social distancing. Additionally, the deactivation of fingerprint access was made for all devices with the use of biological identification technology [50]. The most challenging task was to procure emergency and alternative medicines, assessing the drug shortage, and reserving repurposed drugs for COVID-19 treatment [6]. Proper pharmacovigilance to identify and minimize serious adverse effects and drug interactions among the different new COVID-19 drugs has been carried away by the hospital pharmacists. In particular, corticosteroids were used for patients with respiratory problems but not recommended in viral pneumonia [51].

Because of the restricted availability of personal protective equipment (PPE) in most hospitals, the existence of the pharmaceutical services provided by hospital pharmacists often made them vulnerable to infection regularly. For instance, a window-dispensing method was adopted by many community and hospital pharmacies where doors were shut, and helmet was used as a shield to cover the face to ensure adequate pharmaceutical care service in Nigeria [52]. Furthermore, Hospital pharmacists played a crucial role in the enrollment of infected patients for clinical-trial studies [23].

The highlights of hospital pharmacist’s activity in different countries are the following:

### United States of America

In the USA, pharmacists of the American Society of Health Systems pharmacists (ASHP) supported other healthcare providers by enhancing the safe use of medications and improving patient outcome [34]. During the virtual and hospital bedside rounds, they provided medication recommendations, drug information to the healthcare professionals, delivered education, and information to assist the patients with their lifestyle choices upon admission or discharge. Elson et al. published a report involving healthcare professionals and pediatric patients in the hospital and ambulatory pharmacy. According to them, pharmacists have contributed to adequate storage and drug supply by making phone calls not only to outside pharmacies but also to patient families and insurance companies. They conducted virtual rounds by reviewing patient’s profiles in the EMR (electronic medical report) to ensure safety efficacy of medication therapy, carried away formulary and inventory management through emails, conference calls, Microsoft teams, and provided ambulatory care services remotely. Zuckerman et al. also published a report on healthcare professionals where COVID-19 and non-COVID-19 patients in hospital and ambulatory pharmacy setups where the pharmacists worked on staff redeployment and modifications, counsel patients with COVID-19 on suspending therapy due to immunosuppression, and transition of therapy or treatment procedure [53].

### China

The first guidance and protocols for hospital pharmacy procedures were described in the “Recommendations on Hospital Pharmacy Departments Coping with Corona Virus Disease 2019” (2nd edition) prepared by the Beijing Pharmacy Center for Quality Control and Improvement [25]. A study in Northeast China has shown the efficiency of hospital pharmacists in controlling nosocomial infections and medication errors during the COVID-19 pandemic [54]. Hospital pharmacists also provided direct healthcare services at cabin hospitals of Wuhan, China, where mild cases of COVID-19 infected patients were quarantined and treated effectively [49]. During the ongoing pandemic, the pharmacists in Peking University Third Hospital discussed the role of hospital and community pharmacists and the components of pharmaceutical care [55]. Hua et al. and Meng et al. conducted their respective studies on healthcare professionals and COVID-19 patients in the hospital where pharmacists helped in managing drug formulary, purchase, storage and distribution, loading trolleys with critical care drugs, establishing new pharmacy arrangement, procuring equipment, providing drug information to healthcare professionals, etc. Additionally, Tan et al. published an article on patients undergoing warfarin therapy where hospital pharmacists helped in managing warfarin dose adjustment and change of therapy [53].

### Canada

Pharmacists in Public Health Ontario (PHO), Hotel Dieu Shaver Health and Rehabilitation Centre (HDSHRC) started combining the drug administration time for reducing the use of PPE and minimizing exposure between patients and healthcare providers. Pharmacists shared advice on the appropriate use of MDI (metered dose inhalers), nebulizers, and other intra-nasal drugs in COVID-19 patients. They also monitored the proper use of antibiotics. Canadian Society of Hospital Pharmacists (CSHP) introduced an expanded network of educational videos, links, and other resources to COVID-19 specialty hospitals and other collaborative care giving settings [34, 56].

### Australia

Hospital pharmacists in Australia were directly involved with redesigning COVID-19 specific hospital wards and gathering dedicated teams for the treatment. Within a week, they expanded the existing 7 days a week clinical pharmacy facility of ICU and general medical unit to a 24 h service. Society of Hospital Pharmacists Australia (SHPA) started COVID-19 information hub with other pharmacy departments for sharing information and queries for professional development [34].

### Saudi Arabia

Saudi Pharmaceutical Society (SPS) published a report on COVID-19 and its management. During the rudimentary phase of the pandemic in Jeddah at King Abdulaziz University (KAU) and Hospital (KAUH), a COVID-19 patient rounding team consisting of ID (infectious disease) physicians and ID pharmacists was established. Pharmacists, pharmacy technicians, and students all helped in improving pharmacy care services for COVID-19 in the in-patient and out-patient settings [34]. “Drive-through pharmacy” service has been launched in several hospitals of Saudi Arabia where the hospital pharmacist reviews the prescription and ascertains doses just after the doctor gave the prescription through telephone or online consultation and handed medicines through postal delivery [31].

### Qatar

Hamad Medical Corporation (HMC) in Qatar consisted of 12 general and specialized hospitals were led by more than 400 pharmacists and pharmacy technicians. They showed extraordinary dedication and efficiency during the COVID-19 outbreak. Pharmacists also provided drive-through and tele-pharmacy services [34].

### South Africa

The pharmacists of Netcare Hospital Group Ltd., a network of 54 private hospital played an important role by providing services like maintenance and supply of crucial medications for COVID-19 patients, preparing material packs for surgical items, e.g., ventilation packs, catheter packs, admission packs, and medication packs for prompt and continuous supply within wards. Also, pharmacists have monitored the COVID-19-related treatment regimen. A Monitored Emergency Use of Unregistered Interventions (MEURI) study was designed by a pharmacist for safe and effective monitoring of patients receiving off label therapeutics of COVID-19 in a hospital outside the clinical trial, recommended by the WHO and the South African National Department of Health (NDoH) [34, 57].

### Nigeria

Pharmacists at the University of Nigeria Teaching Hospital being an integral part of the newly launched frontline testing team and treatment center played a vital role by prescription filling, monitoring, and managing medication stewardship of antibiotics, immune boosters, and over used anti-viral agents when there was no official treatment regimen for COVID-19 treatment [34].

### Lebanon

Knowledge and preparedness of pharmacists toward COVID-19 pandemic across Lebanon was surveyed by an English-based online questionnaire through social media platforms, e.g., Whatsapp, Linkedin, and Facebook. Results showed participants had more than 90% knowledge about COVID-19, almost 67% were maintaining safety recommendations. Despite facing shortages, increased prices, delayed supply of masks and sanitizers, 50% of hospitals had taken immediate practical steps during the pandemic outbreak [58]. Despite facing economic and political unrest, Lebanese pharmacists have played key roles on the frontlines of the COVID-19 pandemic in community, hospitals, and academic fields since the first case on 21 February, 2020 [59].

### Spain

Spanish Society of Hospital Pharmacy (SEFH) supported research by creating its own Research Committee and taking part in clinical trials. The Spanish Agency for Medicines and Medical Devices (AEMPS), along with the Institutional Review Boards adopted a fast-track review procedure for accelerating approvals for clinical trials of COVID-19 treatment. From 176 recorded clinical trials in the European Union Clinical Register, Spain ranked first by conducting 68 clinical trials, the second was France with 42 clinical trials followed by the UK with 28 clinical trials. Funding is crucial to conduct clinical trials and research and initiative like “Caixaimpulse COVID-19” launched by La Caixa Foundation, in Spain and Portugal supported clinical and translational research projects with a budget of 1.5 million euros for the use of new technologies for the COVID-19 prevention, management, follow-up, and diagnosis [60].

### Miscellaneous

In the UK, pharmacists in the hospital had to work beyond their usual duties including relocation to the intensive care unit, managing and monitoring COVID-19 patient clinical trials. Some UK pharmacists provided support to four African Commonwealth countries through Commonwealth Partnership for Antimicrobial Resistance and Commonwealth Pharmacists Association. Pharmacists also helped in developing COVID-19 medication protocols, identifying therapeutic drug alternatives, assisting with investigational drug administration, etc. [34]. A survey of pharmacists from 16 European countries including Belgium, Czech Republic, France, Italy, Northern Ireland, Switzerland, and Turkey showed that pharmacists in hospital setups helped in adjusting accommodation facilities, maintaining safe staff redeployment, and minimizing transmission, ensuring continuous medication, PPE and disinfectants supply, collaboration with inter personnel, etc. [39].

## Contributions of clinical pharmacists in COVID-19

Clinical pharmacists are specially trained licensed practitioners who provide all types of direct patient care settings by utilizing their in-depth knowledge of medications [61]. Clinical pharmacy services offer an integrative healthcare system that optimizes comprehensive use of medicine for ambulatory and acute care patients by entailing both community and hospital [62]. The downside of COVID-19 exerted a lack of self-management practices and poor medication adherence among patients with chronic diseases [29]. Clinical pharmacists play a pivotal role in developing innovative strategies and formulating work instructions to promote rational use of the drug with the collaboration of other healthcare professionals [24]. Amidst the heat of the COVID-19 pandemic, they provided a wide range of inpatient services including emphasizing dosage; multidrug therapy reviews; providing precise provision of patient assistance; monitoring off-level use of the drug, medication efficacy; or adverse drug reaction; attending clinical rounds; preparing discharge, and proper counseling of patients [63, 64]. Huibo Li et al. [55] summarized the activity of clinical pharmacists during COVID-19 as follows:

- Development of guidelines for the provision of pharmaceutical services
- Create directory and medical news
- Drug assessment based on proper evidence
- Analysis of remote orders and distribution
- Developing personalized treatment strategies
- Providing telehealth consultation and correct medical information to patients

Clinical pharmacists work as a mediator between physicians and patients and keep their knowledge up-to-date on the latest information from federal agencies, such as the Centers for Disease Control and Prevention (CDC) to identify symptoms, carry out elementary screenings, and confirm individuals having relevant epidemiological risk factors [23]. They are also guiding outpatient dispensing services where there is a shortage of medications taken for autoimmune disorders chronically. In addition, they are minimizing the exposure of patients to COVID-19 by offering telemedicine services, transferring a prescription to mail orders, coordinating with external laboratory facilities, and remain in contact with patients receiving treatments in the clinic to address their concerns. Clinical pharmacists are also optimizing patient care by maintaining insurance coordination of self-monitoring equipment and infusion [65, 66]. Telemedicine service involving clinical pharmacists has some advantages including flexibility for scheduling chemotherapy, anticoagulant therapy, chronic pain prescription, and also special care diseases [67].

The services of a clinical pharmacist are not confined to only COVID-19 patients as there is a strain on the number of healthcare professionals. Cancer patients are immune-compromised and the critical environment of the hospital increases the exposure to COVID-19 infection. Pharmacist’s role in guiding cancer patients amidst the current pandemic is of great importance as most cancer patients are needed to visit the hospital for a routine checkup and chemotherapy [62]. Clinical pharmacists have played a significant role in assisting the investigation of various novel experimental agents in controlled studies for the prophylaxis and treatment of COVID-19. They also facilitate patient assessment to ensure the favorable opinion of the Ethics Committee for the emergency use of the investigational new drug through compassionate use protocols [17]. They are also acquainted with the clinical trial management system where they actively co-operate with research sponsors, undertake inventories of research portfolio libraries, and evaluate the safety and efficacy of related trial drugs [68]. Gross et al. pointed out the critical role of clinical pharmacists in interpreting scientific literatures and approved drug information of other disease-states to provide substantial information to the clinicians and repurposed them for COVID-19. Pharmacists are conducting these experimental drug studies and helping drug discovery through sympathetic use protocols. In the USA, clinical pharmacists were actively engaged in the initial phase of remdesivir’s clinical trial where they carried out a study for the clinical assessment process of the patient, kept previous medication history of the patient, participated in clinical decision making, and informed patient consent [35].

Luisetto indicated clinical pharmacist’s association in achieving clinical improvement in many medical teams [17]. Leguelinel-Blache et al. depicted the role of ICU pharmacists providing bundled care services to 1164 patients with severe conditions which concluded a decrease in the length of stay and mechanical ventilation duration for the ICU patients along with the minimization of overall healthcare cost services by 10,840 euros [69]. Chinese clinical pharmacists monitored the action of lopinavir/ritonavir, ribavirin, glucocorticoids, and immunomodulatory drugs closely in the early period of COVID-19 because of their safety issues [70]. Karasneh et al. reported the expertise of pharmacists having good basic disease knowledge on COVID-19 [17]. It is expected that, when the vaccine will be available, clinical pharmacists may administer tests approved by the US FDA (The United States Food and Drug Administration) [37].

We highlighted the prime global activities of clinical pharmacists in Table 2.

**Table 2:** Global activities of clinical pharmacists in COVID-19 pandemic

Continent	Country	Activities
North America	USA [34, 71]	•American College of Clinical Pharmacists (ACCP) made short documentary stories about COVID-19 related practice, information, research, education, and leadership. •Started a new ambulatory care COVID-19 clinic for management of patients after hospital discharge at Ohio, Columbus. •Provided service via telephone calls and collected information by MPN-SAF TSS and DIPSS plus and updated the information to the EMR before patient’s next visit. •A US ID pharmacist from Miami Florida along with other clinical ID pharmacist all over the world started online platform, the IDstewardship.com blog and @IDstewardship social media profiles which are free and open access to over 41000 users. They provided reliable information during this pandemic.
Europe	UK [34]	•Along with Royal Pharmaceutical Society, United Kingdom Clinical Pharmacists Association started training webinars and clinical resource hub and delivered relevant resources for clinical pharmacists. •Helped in policy making, advocacy and national guidance for pharmacists. •Facilitated in COVID-19 clinical trials, intensive care units (ICU).
	France [72]	•Besides taking care of COVID-19 patients, clinical pharmacists were involved in managing, analyzing and answering questions on an online Q&A hub (https://sfpt-fr.org/covid19) formed by French Society of Pharmacology and Therapeutics.
Asia	China [70]	•Provided updated treatment protocols, drug information, new drug therapy report, and helped the physicians as frontline caregivers by managing adverse drug reactions, dosing, drug-drug interactions, etc. •Gave extra attention to particular patient population during the pandemic, e.g., elderly, pregnant, children, patients with previous chronic illness. •Monitored drug-drug interactions for safety concerns such as lopinavir, ritonavir, tocilizumab, interferon, glucocorticoids, and immunomodulatory drugs. •Provided pharmacovigilance research and remote virtual patient counseling.
	Saudi Arabia [34]	•Saudi Society of Clinical Pharmacy (SSCP) published an opinion paper on Pharmacists roles and responsibilities during epidemics and pandemics in Saudi Arabia. •A team of clinical pharmacists was involved in analyzing, publishing, updating and creating treatment protocols for COVID-19 according to the dynamic changes of the pandemic situation.
	Qatar [34]	•A clinical pharmacist’s team dedicated as the frontline responders for the new established COVID-19 hospitals situated across Qatar and provided clinical interventions and partook in daily rounds.
	Thailand [73]	•Clinical pharmacist team provided tele-monitoring service on daily basis for every patient by using physician order entry system. •Co-operated with physicians and nurses for handling critical cases of COVID-19.
	Lebanon [58]	•Clinical pharmacists along with the hospital multidisciplinary teams played crucial and unique roles in this pandemic. At American University of Beirut Medical Center (AUBMC), the ID pharmacist analyzed the information and research papers to establish a local clinical guideline for management of COVID-19. •As frontline caregivers the clinical pharmacists performed therapeutic monitoring of COVID-19 patients and reviewed daily medication regimen
	Pakistan [74]	•Promptly adapted the national COVID-19 treatment guidelines and provided healthcare to COVID-19 as well as non COVID-19 patients. •Worked beyond their usual duties including, prescribing support, patients medication chart review, ward rounds, and counseling. •Took part in reviewing new updated guidelines and treatment regimen
Africa	South Africa [57]	•South African Society of Clinical Pharmacy (SASOCP) published a practice guide for clinical pharmacists presenting the role of clinical pharmacist in COVID-19
	Nigeria [34]	•Clinical Pharmacy Association of Nigeria (CPAN) started webinars about COVID-19 and its effect on daily work life. •Department of Clinical Pharmacy, The University of Nigeria, Nsukka, Enugu was a part of the National Scientific Advisory Committee for verifying newly established COVID-19 cure. •Pharmacists from the Department of Clinical Pharmacy, in the Africa Resource Centre Nigeria Hub helped to design receptive health supply chain systems for effective distribution of PPE and medicine for different states.
Oceania	Australia [34]	•Clinical pharmacists with the National COVID-19 Clinical Evidence Taskforce developed national, evidence based guidelines for the clinical care of COVID-19 patients. •Provided clinical reviews for outpatients via Telehealth and remote counseling.

## Contributions of industrial pharmacists and pharmaceutical companies in COVID-19

The outstanding achievements of industrial pharmacists are getting unnoticed. They are the prime reason behind smooth services provided by other healthcare professionals. In the initial COVID-19 period, the bulk production of drugs was hampered and drug shortage events were observed globally as China and India—the main suppliers of active pharmaceutical ingredients (APIs) were unable to export products due to increased domestic API consumption. Moreover, the medicines used for COVID-19 treatment in hospitals including respiratory drugs and painkillers experienced an increase from 100% to whopping 700% since the outset of the pandemic [75]. To meet this increased demand for medicines and medical devices, the industrial pharmacists had to work around the clock to scale up the manufacturing process that ensures smooth supply of proper medicines, surgical items, disinfectants, immune-modulators, and other related medicines. For instance, the production of surgical masks in Taiwan was rapidly increased to 13 million masks per day to allow the Taiwanese population to get the individual allowance of 10 masks every 2 weeks [41]. During clinical research, industrial pharmacists were often responsible for determining the ethical aspects and using the industry’s successful tools [6].

Report showed 72% of confirmed vaccine projects had been being led with the aid of the private/pharmaceutical industry while the remaining 28% are managed by universities, the public sector, and other non-profit companies [76]. From decades of experience gained with previous epidemics, industrial researchers are utilizing their deep scientific knowledge to identify the potential of existing treatments for research and development. European industrial pharmacists are partnering with academics, neighboring and global companies, and life sciences sectors to find a way to save the world from further destruction of the ongoing pandemic [77].

The contributions of pharmaceutical companies during COVID-19 pandemic are highlighted in Table 3 [77].

**Table 3:** The contributions of pharmaceutical companies during COVID-19 pandemic

Area	Company	Activities
Treatment development	Alexion	Developed a potential medicine against COVID-19 and it is currently on a phase III clinical trial in hospitalized adult patients.
	AstraZeneca	R&D teams have promptly started a project with the aim of discovering monoclonal antibiotics that will be effective against novel coronavirus.
	Johnson & Johnson	J&J with collaboration are screening existing molecules and also new active compound against COVID-19 virus.
	Pfizer	Conducted a preliminary evaluation of some previously developed antiviral compounds and that suppressed the replication of similar coronaviruses to SARS-CoV-2 in cultured cells.
	Novartis	Aimed for initiating a phase-III clinical trial of canakinumab among pneumonia patient caused by novel coronavirus.
	Amgen	Collaboration with biotechnology company to design and develop antibodies that may become successful to neutralize or inhibit the SARS-CoV-2 virus.
Vaccine development	Seqirus	Working with the University of Queensland to support the CEPI-funded COVID-19 vaccine program based on molecular clamp technology.
	Sanofi	Work is underway to use a previously developed SARS vaccine with their own recombinant DNA technology.
	Pfizer	Pfizer and BioNTech have teamed up to develop a BioNTech mRNA vaccine candidate to prevent COVID-19 infection.
	Johnson & Johnson	Expanding partnerships with the Advanced Biomedical Research and Development Authority (BARDA) and establishing new partnerships with Beth Israel Diaconis Medical Center (BIDMC) to accelerate the development of potential novel coronavirus vaccines.
	MSD	MSD announced three scientific initiatives, including two agreements with Themis Bioscience and IAVI to develop a potential SARS COVID-19 vaccine, and a partnership with Ridgeback Bio leading the development of new antiviral therapeutics.
Diagnostics	AstraZeneca & GSK	GSK, AstraZeneca, and Cambridge collaborated to set up a new test lab at the University’s Anne McLaren lab. GSK and AstraZeneca are working together to provide process optimization assistance to UK National Test Centers in Milton Keynes, Alderley Park, and Glasgow for COVID-19, and to provide expertise in automation and robotics to support national test systems.
	Roche	They announced a new antibody test. It is an in vitro test that uses human serum and plasma taken from a blood sample to detect antibodies and determine the body’s immune response to SARS-CoV-2.
	Takeda	Working with other pharmaceutical authorities and companies through Europe’s Innovative Medicines Initiative (IMI), we leverage our joint expertise to diagnose COVID-19 and develop inhibitors to prevent future outbreaks.
Helping NHS	Alexion	The Alexion Charitable Foundation has donated funds to three non-profit partner organizations, including the WHO COVID-19 Unity Fund.
	AstraZeneca	Offered 9 million masks to support healthcare professionals around the world in response to the COVID-19 pandemic.
	Johnson & Johnson	Donated £1 million, to London College of Hygiene and Tropical Medicine to guide public opinion on current and potential future actions to curb and treat COVID-19, 30,000 bottles of important toiletries and hand cream worth £250,000 for distribution to healthcare workers, NHS expert.
	Novo Nordisk	Working with the NHS, we have opened a new helpline to support diabetics during the outbreak of COVID-19.
	Takeda UK	Donated over £100,000 to patient organizations and charities in urgent need.

## Significance of pharmacists’ inter-professional and intra-professional collaboration in COVID-19

From an ethical and professional perspective, inter-professional and intra-professional collaboration is significant to reduce inequalities and optimize the highest level of healthcare services. This collaboration becomes more critical amidst public health crisis like the current COVID-19 pandemic [17]. This pandemic is not the first health crises for the pharmacists to fight. A study in Saudi Arabia during MERS showed that pharmacists collaborating with primary healthcare providers in the treatment of severely ill condition patients played an enormous role in the clinical decision-making and medication management system. In that case, the pharmacist’s knowledge of disease (88.9%) was second-highest below the physicians (95.7%) and had shown a positive attitude (94.4%) toward the crisis [78]. Pharmacists are serving as frontline fighters alongside physicians, nurses, and non-technical staff to ensure better health outcomes [14]. The contributions of pharmacists in the correctional home of low- to middle-income countries during COVID-19 were also exemplary. Their activities ranged from providing treatment for minor diseases, disseminating the right information, preventing substance use disorder (SUD) to collaborating physicians of medical clinics in order to ensure better treatment of the inmates [79]. Klepser and his research team previously demonstrated the importance of the physician and pharmacist’s collaborative efforts for timely treatment of influenza-like illness (ILI) [17]. Through remarkable collaboration, pharmacists of the UK (UK) and Pakistan have compiled 10-step protection guidelines both in English and Urdu languages to reduce community transmission among the frontline pharmacists [38]. Additionally, industrial pharmacists of many European biopharmaceutical companies are working collaboratively with utmost dedication across the healthcare communities, utilizing leading scientific technology, manpower, and resources with concerted effort to find the best possible solution to combat COVID-19 [80]. The vigilant contact and integrated collaboration of industrial pharmacists with hospital pharmacists are of utmost importance to control the drug shortage events [6]. In the COVID-19 clinical trial management system, pharmacists are working in collaboration with physicians, research sponsors, and local investigational drug services. It is anticipated that, pharmacists will work in collaboration with health economists, epidemiologists, biostatisticians, policy-makers to evaluate the total medical and healthcare expenditures in the near future [17].

## Global recognition of pharmacists in COVID-19

The role of pharmacy professionals vary across countries and their expertise is underestimated in some situations [81]. Canadian national survey of 2015 reported that pharmacists gained the recognition of 64.6% public, 57.4% nurses, and 38.9% physicians as vaccine providers. In the same year in Australia, almost 95% of the patients were satisfied with the vaccination services provided by the pharmacists and were willing to return to the pharmacists in the future. Additionally, 97% of patients would recommend the service to others [12]. In a national survey, the Canadian Pharmacist Association (CPhA) found out 73% of pharmacists reporting an increase in verbal abuse and other form of harassment by patients since the beginning of the COVID-19 crisis. Asian Pharmacists of Chinese descent faced an additional burden of harassment and anti-Asian racism in many countries like the USA, the UK, and France as a result of the COVID-19 pandemic [82].

The government of New Zealand and Northern Ireland has acknowledged pharmacists by providing extra remuneration amidst COVID-19, while Canada’s most populace province Ontario excluded pharmacists as part of the frontline workers [37, 83]. In Malaysia, a previous study reported an average of 6.1 medication-related complications for every resident in nursing homes. Pharmacists have a big role to play in curtailing such problems and ensuring the quality use of medicines [84]. But many hospitals in Malaysia have enlisted clinical pharmacists as “non-essential medical staffs” amidst COVID-19. It is disheartening for the pharmacists as the non-essential medical staff was prohibited from entering wards to minimize the risk of COVID-19 infection [85]. In fact, the government of many countries had not included them in the pandemic preparedness setting despite their active responses in previous pandemic situations [29]. The nature of pharmacist’s duties made them exposed to this contagious infection since they are in direct contact with the patients and infection can be developed through pre-symptomatic and asymptomatic individuals. Importantly, several countries like the USA, the UK, Australia, New Zealand, Canada, France, Ireland, South Africa, Spain, and Turkey have recommended the use of PPEs and disposable gloves for pharmacists when dealing with suspected or infected patients. On the contrary, there was no mention of PPE for pharmacy personnel in Belgium [86]. In Pakistan, community pharmacists have confronted transmission risks as the space in pharmacy stores were limited and even in the city’s largest pharmacy, pharmacists were not provided with proper PPE [87]. If they are not provided with proper PPE’s, it will increase their vulnerability to this infection [38, 68]. Sadly, eight community pharmacy staffs died in Italy as they did not have access to PPE and were exposed to the COVID-19 infection [36].

It is also observed that the fear of contracting COVID-19 led many community pharmacy workers to exhibit reluctance in the discharge of duties. Therefore, the safety of pharmacists is also of utmost importance [20]. Even before the emergence of COVID-19, a lack of recognition for pharmacy services was also observed. Pharmacists were not given enough value for their services by other healthcare professionals in the renal multidisciplinary team of a South African tertiary hospital [88]. In Western Australia, community pharmacists delivering vaccination services were also denied receiving any financial remuneration from the government [21]. Onozato et al. pointed out the significance of administrative and political support within healthcare institutions to maintain the quality of clinical pharmacy services [89]. Bauman demonstrated that pharmacists did not get proper acknowledgement during COVID-19 like other frontline fighters. Even though pharmacist’s attributes are well known to physicians, respiratory therapists and nurses, the knowledge does not always get translated to the news media, general public, and politicians [90]. Pharmacists are the heroes behind the scenes who should be addressed and given the recognition they truly deserve.

## Obstacles to pharmacy services during COVID-19 and future prospects

Stress and anxieties are part of normal human reactions in crisis moments. Pharmacists are not beyond these as they had to play a dual role of healthcare givers and retailers [91]. The current pandemic has caused extraordinary and prolonged demands on the whole healthcare system and caregivers [92]. In order to manage the pre-existing community needs, pharmacists had to adapt to the new challenging and dynamic healthcare regulations. They had to deal with the intensified anxiety and stress because of the workloads, risk of infection and transmission, aggressive patients, and financial aspects of the pandemic. Clinical pharmacists were stuck in a catch-22 situation where they wanted to deliver healthcare services to the patients but lacked proper support to deliver healthcare. A study concluded that pharmacists experienced the highest rate of burnout due to excessive workloads compared to physicians and nurses even before the beginning of this pandemic period [60, 93]. Despite appropriate recommendations made by the clinical pharmacists, physician’s reluctance to alter their colleague’s prescriptions adversely affected the role of pharmacists in a multidisciplinary team [94]. During COVID-19, pharmacists in Canada had spent 24% of their shift in dealing with medication shortages which placed a huge burden. Additionally, a USA-based survey study in nine major hospitals across the country indicated fewer patients were interested in seeking healthcare in hospitals due to the fear of infecting COVID-19. Instead, they went to the community pharmacists to receive guidance leading to a strain in the community pharmacist’s role as they had to fill the clinical role to some extent. Besides dealing with technical difficulties, pharmacists also encountered ethical dilemmas in prioritizing one patient over another. This is also detrimental to the mental well-being of a pharmacist. Pharmacists have faced risks and anxieties in their personal lives outside their professional lives because of lack of childcare, chances of infecting other family members, regular social interactions, and leisure time that are adaptive methods to deal with stress [82, 95].

In low- and middle-income countries (LMICs), the healthcare system had already struggled with many multifactorial challenges and barriers like substandard patient care quality, limited access to the world health system, drug shortage, insufficient health professionals, inadequate financial support, and limited budget. The current pandemic has aggravated these crises and challenges. However in LMICs, the core responsibility and duties of pharmacists being limited to drug dispensing, providing medication information and counseling, and monitoring interaction among drugs [96, 97]. For instance, the government of Pakistan provided inadequate attention to the pharmacy sector as a frontline caregiver and regulated community pharmacy as a regular commercial unit [98]. Due to a lack of appropriate practical, clinical research opportunities and knowledge of advanced technology and pharmacy services, fresh pharmacy graduates could not contribute much to this COVID-19 pandemic [99]. A study conducted by the pharmacists in Egypt displayed that Egyptians had confidence on their healthcare system as they immediately implemented intensive and affective measures to combat the pandemic situation [100]. Pharmacists of low- and middle-income countries were also keen on attending online webinars and workshops, following WHO guidelines regularly to understand new strategies to utilize drug reconciliation and patient counseling. The first COVID-19 webinar arranged by Commonwealth Pharmacists Association (CPA) saw the participation of 620 pharmacists from 38 countries where the top five registered countries (Nigeria, Kenya, Malaysia, India, and Pakistan) were from LMICs. Despite facing numerous difficulties, pharmacists (hospital, clinical, and community) of LMICs had come forward as frontline caregivers to manage the increasing challenges and limitations related to the pandemic [16, 83].

However, from the literature review, it is evident that pharmacist has carried away their responsibilities despite many obstacles like supply shortage, rapidly changing policies, recommendations, treatment design, increased burden on the health system, and also difficulties in direct patients-pharmacists communication due to risk of transmission. In many countries, laws and regulations for the pharmacy practice had been immediately adapted which gave the pharmacists their long due scope and opportunities to serve the patients and to demonstrate that their contribution is valuable to the healthcare system [101]. Cadogan and Hughes described frameworks for pharmacists in the UK and Canada, where pharmacists can evaluate and manage patients according to the pre-existed formulary and drug scheduling models for their minor ailments during a time of routine check-ups or rounds if physicians are unavailable or reduced [27]. Meantime, different value-added services (VAS) provided by pharmacists have become popular which can be useful in healthcare services in the future. Drive thru pharmacy, has become widely accepted in the USA, the UK, Malaysia, Taiwan, Jordan, Croatia, Australia, and many other countries. Rolls Royce service, chronic illness card, forward dispensing, one-stop-shops, prescription reminder, home delivery services, the click-and-collect technology, the Hub and Spoke dispensing models, supplementary prescribing, the ATM-style prescription collection point, and independent prescribing models are some of the VAS used in Australia, UK, and many other countries. Some of these services started before the COVID-19 but during the pandemic era became more popular. Digitalization and the use of technology in the pharmacy sector even after the COVID-19 may enable better healthcare services and ensure a healthier life for everybody [102].

## Limitations of the review

This review included only those activities by pharmacists that were performed for managing COVID-19 situations and did not consider the regular activities of pharmacy professionals. All the articles related to our study of interest may not be covered due to not being indexed in the databases we searched or being available at different websites. Besides, the published articles on COVID-19 are increasing on a regular basis and some articles may miss out as they may be available after the established search time period. This review did not explore the quality of the published papers taking into account but aimed to signify the dedication and engagement of pharmacy professionals being a part of the frontline savior team.

## Conclusion

COVID-19 pandemic has not only been a pronounced challenge and burden on the healthcare system that the human race experienced together all over the world but it has also opened up new doors that have changed the structure of the global healthcare system. Adaptation of new situations, a gradual extension of existing knowledge and skills are in a need for all healthcare professionals, especially for the frontline healthcare service providers. The way of providing pharmaceutical care has been entirely changed during this pandemic, and pharmacists all around the world are concerned about the new regulations, systems, and services. Pharmacists are taking more responsibilities to reduce and control this pandemic. They are now included in more engaging positions at healthcare services by many countries emphasizing the importance of pharmacists. In less developing countries, pharmacists are working without proper safety measures and are unable to utilize their full potentials as they are not provided all the support needed. The regulatory affairs, policy-makers, and academics should come forward to design more suitable models to utilize their competence and increase the workflow of the patient care services.

## Data Availability

Not applicable

## Abbreviations

SARS: Severe acute respiratory syndrome
MERS: Middle East respiratory syndrome
SARS-CoV-2: Severe acute respiratory syndrome coronavirus 2
WHO: World Health Organization
PPE: Personal protective equipment
ID: Infectious disease
ICU: Intensive care unit
SUD: Substance use disorder
ILI: Influenza-like illness
CPA: Commonwealth Pharmacists Association
API: Active pharmaceutical ingredients
CPhA: Canadian Pharmacists Association
EMR: Electronic medical report
ASHP: American Society of Health Systems pharmacists
PHO: Public Health Ontario
HDSHRC: Hotel Dieu Shaver Health and Rehabilitation Centre
MDI: Metered dose inhalers
CSHP: Canadian Society of Hospital Pharmacists
SHPA: Society of Hospital Pharmacists Australia
SPS: Saudi Pharmaceutical Society
KAU: King Abdulaziz University
KAUH: King Abdulaziz University Hospital
HMC: Hamad Medical Corporation
MEURI: Monitored emergency use of unregistered interventions
NDoH: South African National Department of Health
SEFH: Spanish Society of Hospital Pharmacy
AEMPS: The Spanish Agency for Medicines and Medical Devices
CDC: Centers for Disease Control and Prevention
US FDA: The United States Food and Drug Administration
LMICs: Low- and middle-income countries
VAS: Value-added services
